# Monitoring Adhesive Joint Integrity Degradation Under Tensile and Fatigue Loading in Aluminum and CFRP by Electrical Impedance

**DOI:** 10.3390/s26113446

**Published:** 2026-05-29

**Authors:** Shun-Hsuan Huang, Chow-Shing Shin

**Affiliations:** Department of Mechanical Engineering, National Taiwan University, No. 1, Sec. 4, Roosevelt Road, Taipei 10617, Taiwan; d13522010@ntu.edu.tw

**Keywords:** adhesive joint, single-lap joint, structural health monitoring, electrical impedance measurement

## Abstract

Adhesive joints are widely used in structural applications. However, they are susceptible to degradation under service loads and adverse environmental conditions, leading to eventual catastrophic failure. Thus, the advancement of monitoring tools that can deliver real-time data on the deterioration of adhesive joints is crucial for enhancing the reliability of structures. This study investigated the feasibility of using electrical impedance responses to monitor integrity degradation under tensile and fatigue loading in single-lap adhesive joints in aluminum alloy and carbon fiber-reinforced polymer (CFRP) specimens. Previous works on electrical impedance monitoring of adhesive joint integrity invariably employed conductive adhesives. Theoretical considerations based on the concept of a capacitive system indicate that electrical impedance monitoring may still be feasible even if the joint is non-conductive. This has important implications as it suggests that the structural health of many existing ordinary adhesive joints may be amenable to impedance-based monitoring. To test this possibility, neat epoxy adhesive joints without the addition of conductive constituents were fabricated with aluminum and composite adherends. The specimens were subjected to tensile and fatigue degradation while the impedance responses under different excitation frequencies were monitored. The results showed that impedance monitoring is insensitive for detecting damage during tensile failure because the onset of debonding that produces a detectable impedance change occurs too close to the unstable final failure. For fatigue cycling, debonding developed at an early stage and evolved in a stable manner, and the impedance gradually increased with the number of fatigue cycles, reflecting the development of fatigue damage. These findings indicate that impedance-based monitoring on non-conductive adhesive joints has strong potential for tracking structural integrity degradation, particularly for fatigue loading.

## 1. Introduction

Adhesive joints offer significant advantages over conventional joining techniques such as mechanical fastening and welding, as they alleviate stress concentration, maintain a smooth surface, and reduce the weight overhead of bolts or rivets [1]. In recent years, adhesive joints have gained prominence across various applications, including the aerospace industry, automotive systems, and wind turbine blades [2,3,4]. These joints frequently encounter complex service conditions and fluctuating loads, including static tension, cyclic fatigue, and adverse environmental conditions [5,6]. These exposures may precipitate joint deterioration and ultimate failure. Damage to the adhesive layer and debonding at the joint are frequently difficult to detect in their early stages and can serve as precursors to subsequent catastrophic structural failure. Thus, it is essential to devise means for monitoring the structural integrity of adhesive joints during operation.

Structural health monitoring (SHM) has been developed to provide continuous, in situ assessment of the structural integrity of adhesive joints, thereby reducing maintenance costs and enhancing safety [7]. Current SHM methodologies include surface strain gages [8,9,10], surface-mount [11] and embedded [12,13] electromechanical impedance sensors, fiber Bragg grating sensors [14,15], and electrical conductivity monitoring [15,16,17,18,19,20,21,22,23,24,25,26]. Among these approaches, electrical conductivity-based methods have attracted significant attention for adhesive joint monitoring, as they do not require complex techniques or expensive equipment. Furthermore, unlike other sensor-based methods, this approach provides a global response over the entire joint rather than localized sensing in the vicinity of the sensors.

Direct current (DC) measurements [15,16,18,19,20,21,22,23] have been employed to track damage evolution in bonded interfaces, while alternating current (AC) impedance techniques [24,25,26,27,28] have been used to detect defects and assess changes in bond conditions associated with damage or degradation. However, when applied to practical joint health monitoring, current results still have their limitations: previous studies using electrical methods have mostly focused on conductive adhesive systems, in which conductive constituents, such as graphene [16], carbon black [17], and carbon nanotubes [15,18,19,20,21,22,23], are incorporated to establish a percolated conductive network within the adhesive, and resistance changes were monitored. In the majority of these joint conductivity monitoring works, total resistance changes are measured, and the contributions from damage have not been differentiated from contributions from piezoresistivity, which arise purely from deformation [20,21]. Even if impedance instead of resistance is monitored [24,25,26], changes in the resistive component dominate the impedance changes if the joints are conductive. These results have limited application to the plethora of existing adhesive joints, which are invariably non-conductive. Applications of impedance monitoring on non-conductive bonds were limited to environmental degradation on the joint quality due to humidity [27,28] or salt fog [27] absorption, rather than defect evolution under service loading.

Another limitation in previous studies is the use of artificial defects to mimic degradation. For instance, release films [24] and rubber/wax [26] have been introduced into conductive adhesive joints to simulate defects with specific geometries. Although there are definite defects with known geometries and dimensions in these monitoring approaches, the thickness and dielectric properties of such artificial defects differ considerably from those of real cracks. Moreover, as pointed out in Ref. [26], the existence of an artificial defect will change the damage initiation and progression behavior. Thus, the impedance change characteristics in joints with artificial defects may not represent the progressive evolution of crack initiation and growth in practical joints. Another area that still lacks thorough investigation is the monitoring of joint degradation under cyclic fatigue. DC-based monitoring approaches using conductive networks have been widely applied to evaluate the integrity of adhesive joints under various loading conditions. These include one monotonic tensile loading [16,23] and a few loading–unloading cycles [15,21,23] to failure, as well as cyclic fatigue damage [15,20,22] that involved many thousands to millions of smaller amplitude loading cycles. AC impedance-based monitoring of conductive joints has been applied to tensile loading of lap joints [24,26] and double cantilever beam Mode I fracture [25], while their application to cyclic fatigue-induced degradation in adhesive joints remains relatively limited. Systematic studies on the impedance response of adhesive joints with more realistic evolving defects remain limited, and the qualitative characteristics of impedance variation under mechanical loading, as well as the associated frequency dependence, have not yet been fully clarified. Its application to realistic joint defects generated under simulated service loading is expected to provide further insight into the feasibility of impedance-based monitoring.

An adhesive lap joint between two conductive plates acts as an equivalent parallel-plate capacitor, with the adhesive layer serving as the dielectric. When debonding and cracking evolve, air seeps in, altering the overall dielectric constant. Thus, the electrical capacitance of a lap joint may change as defects initiate and grow. As a result, monitoring the AC impedance of a non-conductive joint has the potential to detect the insidious development of damage. Based on capacitive sensing principles [29,30,31], the feasibility/sensitivity of this kind of monitoring depends on the magnitude of the capacitance change, which in turn relates to the area and thickness of the defect, as well as the relative contrast in the dielectric constants between the adhesive and the defect air gap. However, despite this clear physical basis, the applicability of AC impedance monitoring to realistic adhesive joint damage under mechanical loading has not yet been systematically established. A better understanding of this may shed more light on its potential application to existing ordinary adhesive joints.

To address the limitations of previous studies, the present work investigates the impedance responses of non-conductive adhesive joints fabricated using neat epoxy adhesives, instead of relying on conductive adhesive systems. Instead of unrealistic artificially introduced defects, real damage resulting from tensile and cyclic fatigue loading that simulate service conditions was employed. Impedance development at multiple excitation frequencies is continuously monitored for such damage initiation and evolution. Moreover, both aluminum alloy and composite adherends are systematically examined, empirically and theoretically, to evaluate the influence of adherend materials on the impedance response to further clarify the characteristics of impedance-based monitoring under mechanical loading. Idealized theoretical models were first established to explore the relevant characteristics of capacitive impedance evolution for these two families of adherends. The viability of impedance-based structural health monitoring is assessed by analyzing the impact of various adherend materials and adhesive methods on the recorded impedance responses.

## 2. Materials and Methods

### 2.1. Single-Lap Joint Specimen Preparation

**Figure 1:**
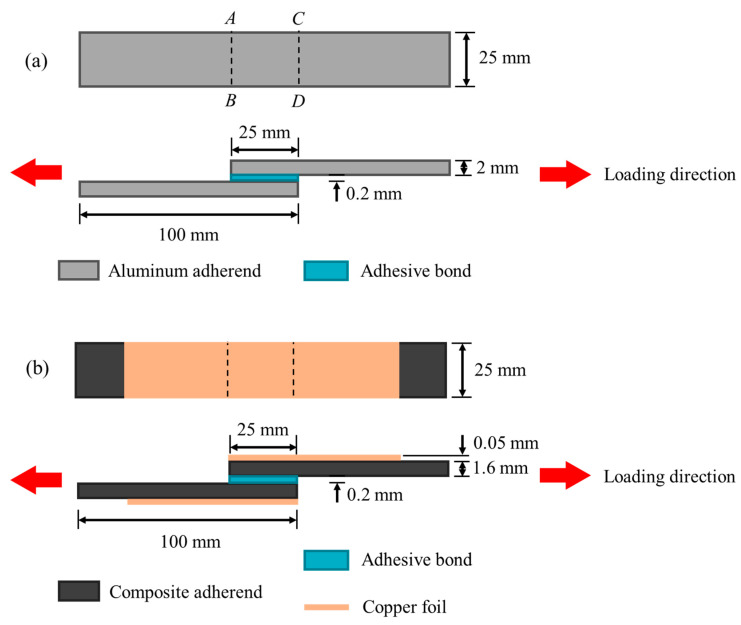
Dimensions and layout of the (**a**) metal-to-metal specimen; (**b**) composite-to-composite specimen.

Both composite-to-composite and metal-to-metal joint specimens were prepared. The metal adherends were machined from 2 mm thick commercial 6061 aluminum alloy sheet (Camellia Metal Co., Ltd., Changhua, Taiwan). The composite laminate adherends were fabricated using a vacuum bag-only process. The laminates have a symmetric hybrid lay-up of unidirectional carbon fiber/epoxy prepreg (Formosa Taffeta Co., Ltd., ECU431, Douliu, Taiwan) and dry unidirectional carbon fiber fabric (Formosa Taffeta Co., Ltd., ECCH, Douliu, Taiwan), with a stacking sequence of [P/P/P/F/P]_s_, where P denotes the prepreg, and F denotes the dry carbon fiber fabric. The latter was employed to provide in-plane flow paths that allow entrapped air and volatiles to evacuate efficiently. The prepreg has a fiber density of 150 g/m^2^ and 37 wt% of epoxy resin. All plies were aligned with fibers in the 0° direction. To establish reliable electrical contact points, a copper foil layer was laminated onto the outermost ply of the laminate. After vacuum degassing at room temperature for 1 h to remove entrapped air between the plies, the laminate was cured using a two-step heating cycle while vacuum was maintained. The temperature was first increased to 120 °C at a heating rate of 2 °C/min and held for 40 min, followed by a second ramp to 150 °C with a 30 min dwell. The laminate was then allowed to cool in the furnace to room temperature. The bonding areas of both the aluminum and composite substrates were carborundum blasted (CES012, Phasic Corp., Yuanlin, Taiwan) before bonding. To remove residual particles after the blasting process, the aluminum adherends were cleaned using an ultrasonic bath (BK-3550, Hanker International Electronics Technology Co., Ltd., Guangzhou, China) in 95% ethanol, and the composite adherends were thoroughly wiped with the same solvent. Schematics of the geometry and dimensions of the single-lap joint specimens are shown in Figure 1.

Neat epoxy resin (Swancor 2261-A/BS, Swancor Industrial Corp., Nantou, Taiwan) was employed for adhesive bonding. After mixing the resin and hardener thoroughly, the prepared adhesive was degassed under vacuum for approximately 10 min to remove any trapped gas bubbles. Throughout the adhesive preparation process, the resin temperature was kept low by immersing it in an ice bath to suppress premature curing. The adhesive application procedure followed the methodology detailed in a previous study [20]. A jig fixture was used to maintain a constant adhesive thickness of 200 μm. The bonded specimens were cured at room temperature for 24 h and then post-cured at 100 °C for 2 h in a laboratory oven (DOS45, Deng-Yng Corp., New Taipei City, Taiwan).

### 2.2. Mechanical Testing

Tensile and fatigue tests were carried out using a servo-hydraulic testing system (MTS 810, MTS Systems, Eden Prairie, MN, USA). The direction of the loading is illustrated in Figure 1. End tabs of suitable thickness are attached to the ends of the specimens to facilitate gripping and ensure that the load line passes through the mid-thickness of the adhesive layer. Two specimens per batch were tested to determine the average batch tensile strength (TS), which subsequently served as the basis for defining the fatigue amplitude parameters. Fatigue tests were conducted under load-controlled sinusoidal loading at 5 Hz. The stress amplitude was set to 5–50% of the average TS for the composite specimens and 6–60% of the average TS for the aluminum specimens, thereby ensuring comparable fatigue lives for each material group.

### 2.3. Impedance Measurement

**Figure 2:**
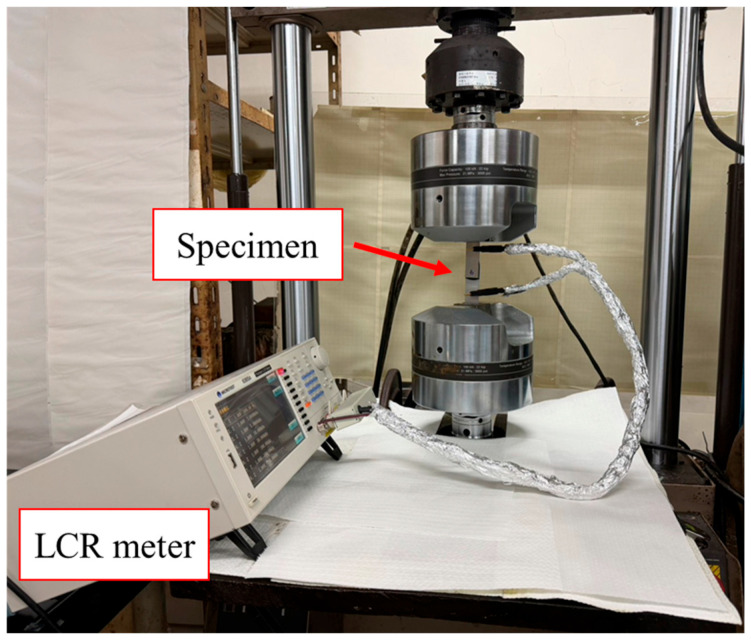
Experimental setup for impedance monitoring during mechanical loading.

Impedance was measured using a precision LCR meter (Model 6365A, Microtest Co., Ltd., Xizhi District, New Taipei City, Taiwan). Specialized coaxially shielded leads that come with the LCR meter allow tight attachment onto the specimen. Before testing, the instrument was calibrated using open- and short-circuit compensation procedures across the full frequency range to subtract the capacitance of the leads, as recommended in the instrument manual [32]. An excitation voltage of 1 V was applied throughout all measurements. A photograph of the actual experimental setup is provided in Figure 2. Impedance was measured at four frequencies (500 Hz, 5 kHz, 100 kHz, and 200 kHz) for both tensile and cyclic fatigue loading. Preliminary monitoring of a load-free virgin specimen on the machine showed no change in impedance with time over a duration of 3.5 h. This precludes any environmental influence on the measured impedance during the tests.

The SHM literature using electrical conductivity [20,21] clearly shows that carbon nanotube-modified epoxy is piezoresistive and that deformation alone will change its resistance. To preclude any similar effect on the impedance, periodic unloading to measure at a constant small load of 50 N was adopted in both the tensile and fatigue tests. The impedance change observed at this near-unloaded state served as a reliable reflection of the accumulated damage in the adhesive joints while being free from the effect of loading/deformation. Further justification for this will be discussed in the Results section.

The percentage change in impedance was used as the primary metric to assess joint integrity and is defined in Equation (1):

(1) $$\%\;Impedance\;Change=\;\frac{\left|Z\right|-\left|Z_{0}\right|}{\left|Z_{0}\right|}\;\times 100\%$$

where |*Z*_0_| is the initial impedance value recorded, and |*Z*| is the instantaneous impedance measured in the course of the mechanical tests at 50 N under the same excitation frequency as |*Z*_0_|. This normalization allowed for direct comparison across specimens and loading conditions.

## 3. Results

### 3.1. Theoretical Capacitive Model of Aluminum and Composite Adhesive Joints

#### 3.1.1. Equivalent Capacitance and Impedance of Aluminum Adhesive Joints

**Figure 3:**
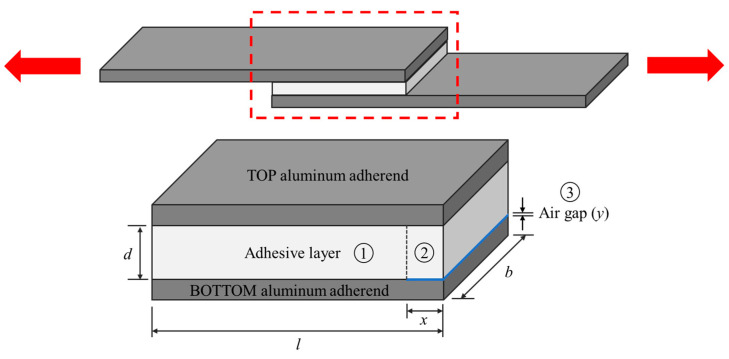
Schematic illustration of the equivalent capacitance model of an aluminum adhesive joint with a debond-induced air gap (blue line). (The red dotted rectangular box is shown in more detail at the lower half of the above figure).

Capacitance depends on the geometry of the conductors and the dielectric [30,31]. When two conductive adherends are bonded by an adhesive layer, the joint can be approximated as a parallel-plate capacitor, with the adherends acting as electrodes and the adhesive layer serving as the dielectric (illustrated not to scale in Figure 3). According to classical capacitor theory, the capacitance of such a system can be expressed as Equation (2):

(2) $$C=\;K\frac{\varepsilon_{0}A}{d}\;$$

*C* is the capacitance in farads (F), *K* is the relative static permittivity (dielectric constant) of the material between the adherends (air = 1 [31], epoxy = 3–6 [33], epoxy/carbon fiber composite = 5~10 [34]), *ε_0_* is the permittivity of free space, with a value of 8.854 × 10^−12^ F/m, *A* is the effective overlap area, and *d* is the separation distance between the two adherends (the adhesive thickness). For a rectangular single-lap joint with width *b* and overlap length *l*, the initial overlap area of the intact bond is *A* = *bl*.

When debonding occurs along the overlap direction, the joint can be divided into two regions along the length direction: an intact bonded region ① and a debonded region ②. Let the debond length be *x*. Therefore, the intact bonded region has a length *l* − *x*. Since these two regions experience the same potential difference between the conductive adherends, they are electrically equivalent to capacitors connected in parallel. The total capacitance of the joint can therefore be considered as a combination of three components. The first component corresponds to the intact bonded region (① in Figure 3), where the dielectric medium remains solely the adhesive. In the debonded region, however, an air gap of thickness *y* is introduced, where *y* is assumed to be small and constant and occurs at the adhesive-adherend interface for simplicity in the present model. Thus, the debonded region consists of two dielectric layers stacked along the thickness direction: a remaining adhesive layer with thickness *d* (② in Figure 3) and an air layer with thickness *y* (③ in Figure 3). Because these two dielectric layers are stacked along the thickness direction, they behave as capacitors connected in series. The derivation is unaffected if cohesive failure occurs instead of the adhesive failure assumed. Accordingly, the total capacitance of the joint, *C*_*total*_, is obtained by summing the capacitances of the intact bonded region and the equivalent capacitance of the debonded region, which can be expressed as Equation (3):

(3) $$C_{total}=\;\frac{\varepsilon_{0}K_{adhesive}b(l-x)}{d}+\frac{\varepsilon_{0}bx}{\frac{d}{K_{adhesive}}+\frac{y}{K_{air}}}\;$$

The initial capacitance for the intact joint, *C*_0_, can be obtained by setting *x* to zero in Equation (3). The impedance magnitude is inversely proportional to the capacitance. The impedance |*Z*| of this system is:

(4) $$\left|Z\right|=\;\frac{1}{\omega C}\;$$

where |*Z*| is the impedance, *ω* is the angular frequency of the applied alternating current (ω = 2π*f*, with *f* being the excitation frequency) and *C* is the equivalent capacitance of the adhesive joint.

The variation in impedance induced by debonding can be quantified using a normalized impedance change and is expressed as the percentage impedance variation as defined in Equation (5):

(5) $$\%\left|Z\right|=\;\left[\frac{\frac{K_{adhesive}l}{d}}{\frac{K_{adhesive}(l-x)}{d}+\frac{x}{\frac{d}{K_{adhesive}}+\frac{y}{K_{air}}}}-1\right]\times 100\%$$

#### 3.1.2. Equivalent Capacitance and Impedance of Composite Adhesive Joints

**Figure 4:**
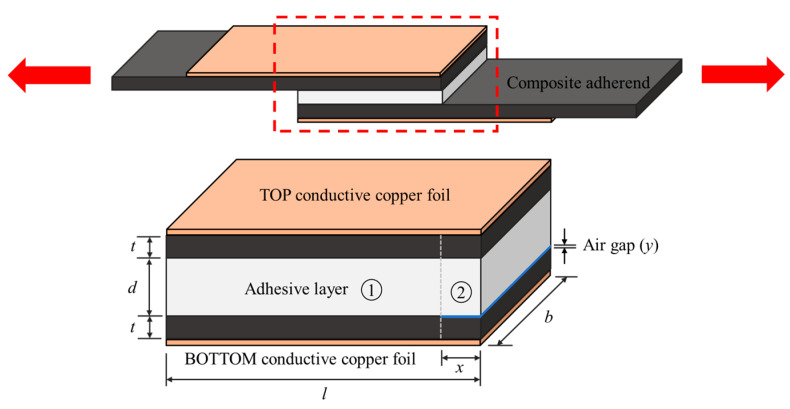
Schematic illustration of the equivalent capacitance model of a composite adhesive joint with a debond-induced air gap (blue line). (The red dotted rectangular box is shown in more detail at the lower half of the above figure).

For adhesive joints with composite adherends, copper foils were used as electrodes and were placed on the outer surfaces of the adherends. The electrical configuration differs from that of an aluminum joint and is illustrated schematically, not to scale, in Figure 4. The dielectric medium between the electrodes consists of multiple layers, including the top composite adherend, the adhesive layer, and the bottom composite adherend. Since these dielectric layers are stacked along the thickness direction, they are electrically equivalent to capacitors connected in series. Let *t* denote the thickness of each composite adherend and *d* denote the adhesive thickness. For a rectangular single-lap joint with width *b* and overlap length *l*, the initial overlap area of the intact bond is *A* = *bl*.

When debonding occurs along the overlap direction, the joint can be divided into two regions along the length direction: an intact bonded region (① in Figure 4) and a debonded region (② in Figure 4). Let the debond length be *x*. The intact bonded region has a length *l* − *x*. Since these two regions experience the same potential difference between the copper electrodes, they are electrically equivalent to capacitors connected in parallel. In the debonded region, the dielectric structure changes due to the formation of an air gap of thickness *y*. The dielectric stack in this region consists of four layers arranged along the thickness direction: the top composite adherend, the adhesive layer with thickness *d*, the air gap with thickness *y*, and the bottom composite adherend. This is electrically equivalent to a series arrangement of capacitors. Thus, the total equivalent capacitance of the composite adhesive joint can be obtained by summing the capacitances of these two regions and expressed as Equation (6):

(6) $$C_{total}=\frac{\varepsilon_{0}b(l-x)}{\frac{2t}{K_{composite}}+\frac{d}{K_{adhesive}}}+\frac{\varepsilon_{0}bx}{\frac{2t}{K_{composite}}+\frac{d}{K_{adhesive}}+\frac{y}{K_{air}}}\;$$

Similar to the aluminum adhesive joint, the system’s impedance magnitude is inversely proportional to the equivalent capacitance. Therefore, the impedance variation induced by debonding, quantified using the percentage normalized impedance change, can thus be expressed as Equation (7):

(7) $$\%\left|Z\right|=\;\left[\frac{\frac{l}{\frac{2t}{K_{composite}}+\frac{d}{K_{adhesive}}}}{\frac{l-x}{\frac{2t}{K_{composite}}+\frac{d}{K_{adhesive}}}+\frac{x}{\frac{2t}{K_{composite}}+\frac{d}{K_{adhesive}}+\frac{y}{K_{air}}}}-1\right]\times 100\%$$

#### 3.1.3. Comparative Analysis of Theoretical Impedance Response in Aluminum and Composite Adhesive Joints

**Figure 5:**
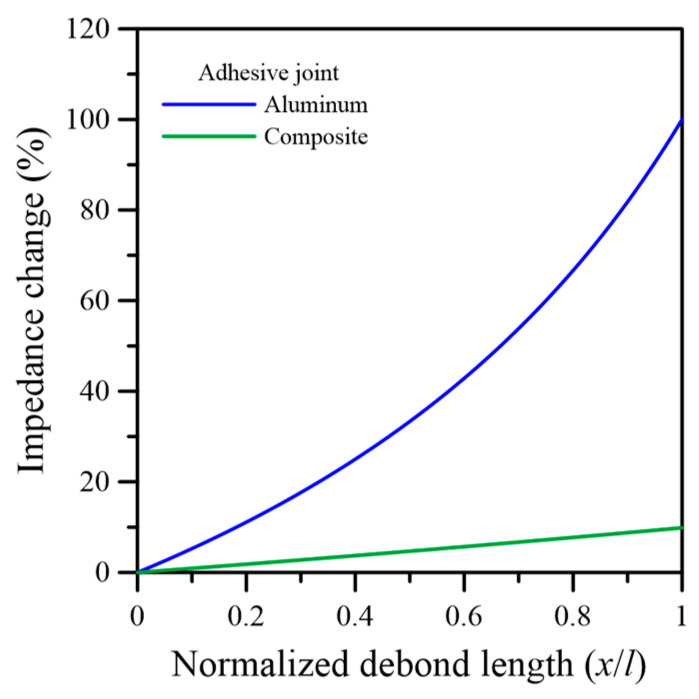
Theoretical variation of normalized impedance changes for the aluminum and composite adhesive joints as a function of normalized debond length.

To further elucidate the differences in impedance response between aluminum and composite adhesive joints, the theoretical relationship between the normalized percentage impedance change *%*|*Z*| and the normalized debond length *x*/*l* is presented in Figure 5 for both cases. The calculation is based on Equations (5) and (7), using the relative static permittivity values from the literature reported in Section 3.1.1; *y* = 0.05 mm is used as an example.

As shown in Figure 5, *%*|*Z*| increases monotonically with increasing *x*/*l* for both types of joints, indicating that impedance is inherently sensitive to the progression of debonding. However, a pronounced difference in both magnitude and trend is observed between the two systems. For the aluminum adhesive joint, the impedance change exhibits a strongly nonlinear increase, with a gradual rise at small *x*/*l* values followed by a rapid escalation as *x*/*l* approaches unity. This behavior suggests that the sensitivity of the impedance to damage increases significantly during the later stages of debond propagation. The nonlinear growth is attributed to the substantial reduction in effective capacitance caused by the extension of the air gap, which directly dominates the electrical response due to the simple dielectric configuration of the aluminum joint. In contrast, the composite adhesive joint exhibits a much smaller impedance increase, with *%*|*Z*| remaining relatively low across the entire range of *x*/*l* and showing an approximately linear trend. Even when *x*/*l* approaches unity, the impedance change is limited to a modest increase compared with that of the aluminum joint. The comparison clearly demonstrates that both systems follow the same fundamental mechanism, yet the impedance response due to damage development is significantly less sensitive in composite joints than in aluminum joints. It is observed that the impedance change in composite joints is approximately one order of magnitude (~10 times) lower than that in aluminum joints. This reduced sensitivity can be attributed to the presence of composite adherends, which introduce additional dielectric layers in series with the adhesive layer. These layers effectively increase the overall dielectric thickness and dilute the influence of the air gap, thereby attenuating the change in equivalent capacitance. By reasoning along this line, it may be inferred that a thinner bond line and/or thinner composite laminate adherends will alleviate the dilution effect and should lead to better sensitivity of this technique.

#### 3.1.4. Limitations of the Above Models

It should be noted that the air-gap thickness is assumed to be constant along the whole debonded length and across the whole specimen width in the present model. In reality, early-stage defects were initiated at localized positions and only spread across the whole width at a later time. Even then, the crack front is unlikely to be uniform across the width. Additionally, as the crack opening is wedge-like, the air-gap thickness varies along the debond length instead of staying constant. Therefore, the present highly idealized model should be regarded as an approximation that captures the dominant trend of impedance variation with debond growth, rather than a fully quantitative predictive tool. Despite these limitations, it provides valuable physical insight into the relationship between interfacial damage and electrical response and serves as a useful framework for interpreting the experimental observations. Another point to be observed from the model is that at the same debond length, the air-gap thickness probably increases with loading. According to the present simplified model, this will increase the capacitive impedance. This reiterates the importance of measuring at zero load or a fixed low load in order to quantify damage without introducing additional but irrelevant impedance changes due only to load/deformation.

Although there is a frequency term in the impedance *Z* (Equation (4)), it is absent in the above expressions for *%*|*Z*| (Equations (5) and (7)) as it is canceled out when the percentage change in impedance is computed (Equation (1)). However, it should be noted that %|*Z*| is not truly frequency independent. Frequency dependence remains indirectly through the dielectric permittivity, which tends to drop with the frequency of the applied AC. This is due to the inability of the alternating alignment of the electric dipoles in the dielectric to catch up with the rapidly changing polarity of the electric field [35]. This frequency dependency is more marked with the epoxy adhesive and the composite [36,37,38] and is negligible with air. As a result, the contribution of the air gap, or defect, to the total impedance will become smaller at higher measuring frequencies. Thus, the %|*Z*| for the same defect is expected to be smaller at a higher frequency.

### 3.2. Impedance Monitoring Under Tensile Loading of Aluminum and Composite Single-Lap Joints

**Figure 6:**
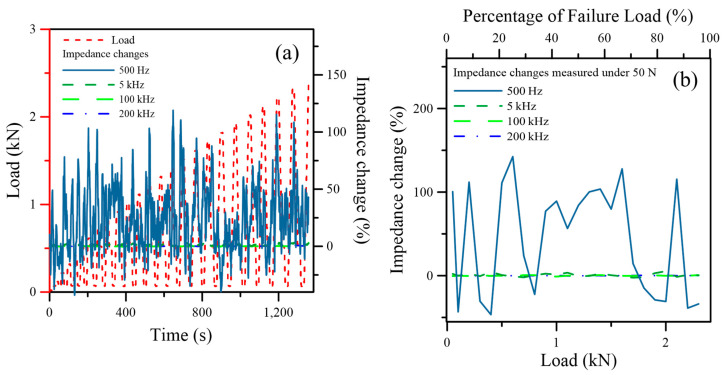
Typical impedance changes at four excitation frequencies in aluminum adhesive lap joints under tensile loading. (**a**) During the course of progressively increased loading and periodic unloading, (**b**) impedance changes were measured at periodic unloading to 50 N.

Figure 6 presents the typical impedance change of the adhesive aluminum joint under tensile loading at four excitation frequencies. Figure 6a displays the percentage impedance change during the course of loading. As noted before, the %|*Z*| includes contributions from joint deformation under load as well as possible damage in the joint. To isolate the effect of damage, impedance measurements were made after periodic unloading to 50 N, and the corresponding %|*Z*| are presented in Figure 6b. In both figures, it can be seen that at an excitation frequency of 500 Hz, the signal is plagued by significant noise and exhibits pronounced haphazard fluctuations without showing a well-defined trend. At 5 kHz and above, no observable change is recorded throughout the test. Excessive noise in the 500 Hz signal is believed to come from an unfavorable signal-to-noise ratio. The excitation voltage is fixed at 1 V. At low frequency, the impedance is very high, so the measured current is extremely small and more prone to noise, resulting in an unfavorable signal-to-noise ratio. The signal-to-noise ratio improved considerably when the excitation frequency increased to 5 kHz and above. Similar responses have also been observed in the tensile testing of adhesive joint specimens without artificial defects by Kim et al. [24]. Impedance-based monitoring is ineffective for assessing the integrity during tensile failure for an aluminum single-lap joint with epoxy adhesive.

**Figure 7:**
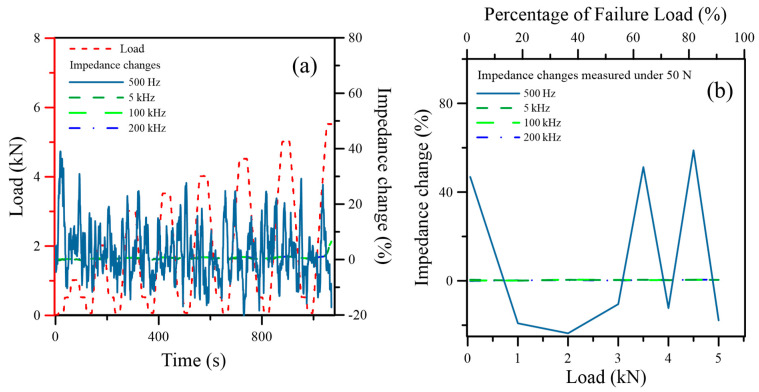
Typical impedance changes in the composite single-lap joint measured at four excitation frequencies under tensile loading. (**a**) During the course of progressively increased loading and periodic unloading, (**b**) impedance changes were measured at periodic unloading to 50 N.

Figure 7 illustrates the impedance response of the composite adhesive joint subjected to tensile loading at four excitation frequencies. As shown in Figure 7a, the %|*Z*| recorded under 500 Hz fluctuates irregularly and does not display a consistent variation with the applied load. At 5 kHz and above, no observable change is recorded until the end of the test, where %|*Z*| seems to rise just before the final failure sets in. A similar behavior is observed during periodic unloading to 50 N, as presented in Figure 7b. The 500 Hz impedance signals remain highly scattered and dominated by random noise, while higher frequency signals showed no change. The rise at the end of the test is not exhibited, as failure occurred before unloading to 50 N had the chance to take place. Again, as in the case of the aluminum joint, impedance-based monitoring is not suitable for monitoring the tensile integrity of a composite single-lap joint bonded with neat epoxy adhesive.

The reason that the impedance monitoring for tensile damage is ineffective may be that the debonding defect under tensile loading occurs only at a very late stage, immediately before unstable failure. Aluminum epoxy lap joint damage monitoring with fiber Bragg gratings indicated that the fiber sensors started to detect joint damage under tensile loading only beyond ~90% of the failure load [39]. Sam-Daliri et al. also observed, in an intact low conductivity epoxy joint with 6 wt% of carbon nanotube, that resistance started to show an observable increase at 95% of the failure load [26]. Liquid penetration marking of the tensile damage of the adhesive lap joint of carbon fiber/epoxy composite showed no sign of debonding at 92% of the failure load [15]. According to the capacitive model outlined in Section 3.1, a change in the joint impedance requires the formation of a debond-induced air gap. If such debonding develops only near the final unstable failure, the impedance change will show no response during loading, and a meaningful indication close to failure may easily be missed when instability sets in quickly.

### 3.3. Impedance Monitoring Under Fatigue Loading of Aluminum and Composite Single Lap Joints

**Figure 8:**
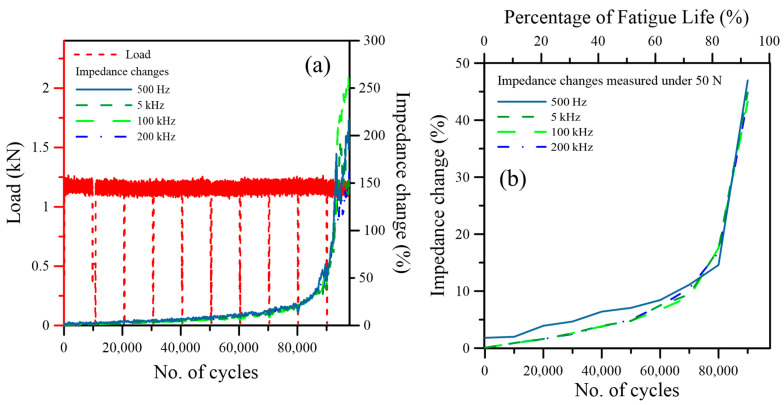
Typical impedance changes in the aluminum adhesive joint measured at various excitation frequencies under fatigue loading. (**a**) Impedance changes in the course of cyclic loading, (**b**) impedance changes measured during the periodic unloading every 10,000 cycles.

Figure 8 presents the impedance change in aluminum non-conductive adhesive joints under fatigue loading. Figure 8a shows the impedance change during the whole fatigue test, including periodic unloading every 10,000 cycles. The impedance signals at all four frequencies exhibit a consistent increasing trend with cycling, which corroborates the characteristic progressive joint degradation during fatigue. The increase is gradual before ~80,000 cycles, and different frequency signals are indistinguishable from one another. Afterward, the increase accelerated. Beyond ~90,000 cycles, the impedance curves at different excitation frequencies began to diverge, and the maximum change reached is above 200% at specimen failure. This behavior can be explained based on the mechanism discussed in Section 3.1. As damage accumulates and debonded air gaps are progressively introduced, the reduction in effective permittivity becomes more pronounced, leading to observable differences among frequencies. The impedance changes measured every 10,000 cycles under 50 N are shown in Figure 8b. The %|*Z*| values from 5 kHz to 200 kHz are indistinguishable from one another and increase smoothly with the number of cycles. This behavior is consistent with the reduction in effective capacitance caused by the formation of air gaps as damage develops. Although the 500 Hz signal is prone to noise, it still reflects the overall trend of damage accumulation.

The liquid penetrant marking technique [15,20] showed that the debonding developed gradually during early cycling at localized positions at the longitudinal edges (AB, CD in Figure 1a) of the joint. In the later stage, the delamination crack spread across the whole specimen width and evolved to a marked length in the load direction. The amount of debonded area increased linearly with the number of fatigue cycles elapsed [15,20]. The increasing trends of %|*Z*| shown in Figure 8 are consistent with progressive fatigue degradation at the Al–epoxy interface and, in fact, are in reasonable agreement with the theoretical predictions in Section 3.1 as exemplified in Figure 5. Note that the evolution of the debonding defect under fatigue is different from that under tensile failure for two reasons. Firstly, with a sufficient number of cycles of repetitive cyclical loading, defects can be initiated and grow even at a small load. Secondly, as the maximum load involved in fatigue testing was typically a fraction of the tensile strength (50–60% in the current work), unstable failure will not set in until the defect grows to a considerable size. These differences are probably the underlying reason that the impedance monitoring of damage evolution in an adhesive joint is effective for fatigue but not for tensile failure.

**Figure 9:**
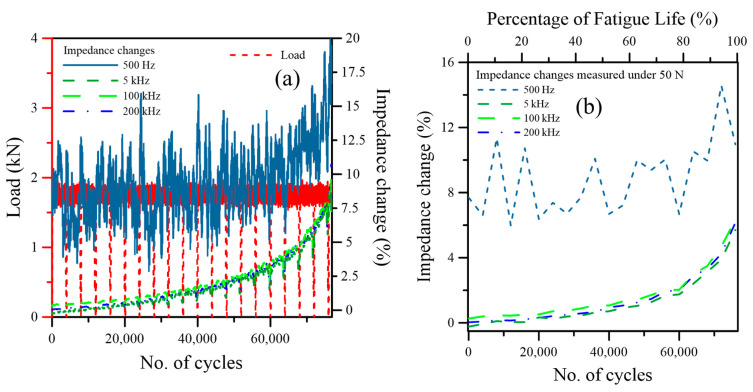
Typical impedance changes in the composite adhesive joint measured at different excitation frequencies under fatigue loading. (**a**) Impedance changes during fatigue loading, (**b**) impedance changes were measured during periodic unloading every 4000 cycles under 50 N.

Figure 9 illustrates the typical percentage impedance change of non-conductive composite lap joints subjected to fatigue loading at four excitation frequencies. Figure 9a shows the change throughout the whole fatigue test, including the periodic unloading. The impedance response at 500 Hz is plagued by noise but shows a weak upward trend throughout the cycling process. In contrast, the impedance responses at the remaining excitation frequencies increased steadily as the fatigue cycles accumulated. The observed fluctuations can be attributed to the higher sensitivity of impedance to lower frequency excitations, and so it is more vulnerable to perturbations in dielectric properties and noise. In contrast, higher-frequency responses are relatively more stable and exhibited a definite upward trend. During the unloading to measure impedance at 50 N, it is clear that the impedance dropped and rose with loading and unloading. This is an unambiguous demonstration that loading or deformation alone will change the impedance, and so to monitor the joint integrity, measurement at a fixed low load is recommended to preclude the unwanted interference from deformation. Figure 9b presents the impedance variations measured under 50 N every 4000 cycles. Again, the response under 500 Hz exhibited pronounced fluctuations without a distinct trend; the impedance at the other excitation frequencies showed a very gentle but progressive increase with accumulated fatigue life. The increase in %|*Z*| reflects the gradual damage accumulation within the specimen, despite its low sensitivity. The overall increase in %|*Z*| during the whole fatigue life is much smaller in composite joints compared to aluminum joints. This is consistent with the theoretical analysis shown in Figure 5.

In summary, the above results showed that it is feasible to monitor the joint integrity degradation under fatigue loading through measurement of the impedance change, even though the joint is non-conductive. This technique is more sensitive in metal-to-metal joints and less sensitive in composite-to-composite joints. As the joints are fabricated from neat epoxy resin and are non-conductive, the result implies that the fatigue integrity of existing adhesive joints may be monitored using this technique.

## 4. Conclusions

The feasibility of monitoring joint integrity degradation under tensile and fatigue loading via electrical impedance has been investigated in non-conductive single-lap adhesive joints of aluminum and composite specimens. The main findings and implications of this study are summarized as follows:

- Theoretical analysis indicates that the formation of a debonding air gap reduces the effective capacitance of the joint, leading to an increase in capacitive impedance. The amount of impedance change is governed by the evolution of the debond length and the air-gap thickness, which establishes a basis for integrity surveillance of the joint by monitoring impedance, even if the joint is non-conductive. Theoretical models also imply that the sensitivity is strongly influenced by the adherend dielectric configuration. In particular, composite joints exhibit significantly lower sensitivity than aluminum joints due to the presence of additional dielectric layers. Moreover, it suggests that better sensitivity may be achieved with a thinner bond line and thinner composite laminate adherends.
- The percentage impedance changes at four different frequencies, ranging from 500 Hz to 200 kHz, all exhibited haphazard fluctuations without a well-defined trend in both aluminum and composite joints during tensile testing. Thus, impedance monitoring is ineffective for joint integrity surveillance under overload tensile failure. The reason for this is that the debonding defect occurs only at the very late stage of loading, close to and probably associated with the unstable final failure, leaving insufficient damage evolution for impedance changes to unfold and be detected.
- The impedance measurement under cyclic fatigue loading increased progressively with the number of cycles, reflecting the gradual evolution of joint damage. The progressive nature of fatigue damage enables effective impedance-based monitoring for non-conductive joints. Higher-frequency (5–200 kHz) responses provide more stable indications of damage progression. Aluminum joints exhibited higher sensitivity with eventual impedance change greater than 200%, while composite joints exhibited reduced sensitivity due to additional dielectric layers.
- For practical monitoring applications, an excitation frequency above 5 kHz is recommended to provide a better signal-to-noise ratio. Although the start of a more abrupt increase in impedance is a warning sign of significant integrity degradation, a simple percentage change in impedance cannot be generalized, as the impedance change behavior depends on the adherend materials and the adhesive material and geometry. Calibration on a case-by-case basis is needed. Moreover, the possible influence of environmental factors such as humidity and temperature on the impedance response should be noted, and load-free dummy specimens may be employed as a reference. The effects of a hygrothermal environment and other mechanisms of failure on the current technique are worth investigating.

## Data Availability

The original contributions presented in this study are included in the article. Further inquiries can be directed to the corresponding author.
